# Comprehensive Transcriptomic and Proteomic Profiling of CILD40 with Novel Compound Heterozygous Mutations of *DNAH9*

**DOI:** 10.1007/s43657-026-00318-0

**Published:** 2026-07-28

**Authors:** Siming Kong, Mingshuo Wang, Xuedong Wang, Yakun Liu, Pengfei Wang, Wenyan Song, Ziye Chen, Yuxin Su, Canhong Xiang, Yunfang Wang, Hui Bai

**Affiliations:** 1 Center for Clinical and Translational Science, School of Clinical Medicine, Beijing Tsinghua Changgung Hospital, Tsinghua Medicine, Tsinghua University, Beijing, 102218 China; 2 School of Clinical Medicine, Hepato-Pancreato-Biliary Center, Beijing Tsinghua Changgung Hospital, Tsinghua Medicine, Tsinghua University, Beijing, 102218 China; 3 Precision Hepatobiliary Center, School of Clinical Medicine, Beijing Tsinghua Changgung Hospital, Tsinghua Medicine, Tsinghua University, Beijing, 102218 China; 4 Faculty of Environment and Life, Beijing University of Technology, Beijing, 100124 China

**Keywords:** Primary ciliary dyskinesia, Primary ciliary dyskinesia 40, *Dynein axonemal heavy chain 9*, Compound heterozygous mutations, Transcriptome, Proteomics

## Abstract

Primary ciliary dyskinesia (PCD) is a rare genetic disorder characterized by impaired ciliary motility that leads to respiratory symptoms, laterality defects, and other systemic abnormalities. Despite significant advancements in genetic research identifying over 50 causative genes and enabling genetic diagnosis in approximately 90% of cases, comprehensive phenotypic characterization remains underexplored. We investigated two respiratory asymptomatic individuals (sisters) who exhibited laterality defects in a three-generation family, both of whom harbored novel compound heterozygous mutations (NM_001372.4:c.308del and NM_001372.4:c.11845G > A) in the *dynein axonemal heavy chain 9* (*DNAH9*) gene associated with primary ciliary dyskinesia-40 (CILD40). Structural modeling and western blotting analysis of HEK-293T cells demonstrated that the frameshift mutation abolished DNAH9 stability, whereas the missense mutation disrupted hydrogen bonds, leading to partial protein destabilization. Peripheral blood RNA sequencing revealed extensive dysregulation of axonemal and intraflagellar transport genes, implicating defects in microtubule-based motility in the two affected siblings with biallelic DNAH9 mutations, but not in their heterozygous family members or one unaffected relative. Plasma proteomic analysis of patients with CILD40 identified significant enrichment of pathways related to platelet activation, complement and coagulation cascades. Further comparative analysis with a patient with PCD caused by a CCDC40 mutation (CILD15 subtype) revealed differential signatures in CILD40, highlighting the potential of plasma proteomics for understanding distinct pathogenic mechanisms across PCD subtypes. These findings underscore the critical role of *DNAH9* compound heterozygous mutations in CILD40 and provide new insights into the genetic, transcriptional, and proteomic phenotypic heterogeneity of PCD.

**Supplementary Information:**

The online version contains supplementary material available at https://doi.org/10.1007/s43657-026-00318-0.

## Introduction

Primary ciliary dyskinesia (PCD) is a rare genetic disorder caused by structural or functional abnormalities in the cilia, leading to diverse clinical manifestations, including laterality defects in thoracoabdominal organs and respiratory tract diseases (Lucas et al. 2014). The global prevalence of PCD is approximately one in 7600 people (Ferkol 2024). Over the past decade, next-generation sequencing has accelerated the discovery of PCD-associated genes, with more than 50 causative genes identified to date, containing more than 2000 pathogenic variants. Genetic testing can now confirm approximately 90% of PCD cases; however, the genetic complexity of PCD presents unique challenges for its accurate diagnosis and management, with approximately 30% of cases remaining undiagnosed using current genetic panels (Cant et al. 2024; Fassad et al. 2020; Hashizume et al. 2024). According to the Online Mendelian Inheritance in Man (OMIM) database, PCD comprises 53 subtypes. However, most studies have focused solely on newly identified genes or variants, leaving comprehensive phenotypic characterizations beyond genomic data largely unexplored.

Cilia are microtubule-based, hair-like organelles protruding from the cell surface and are classified into primary and motile cilia based on their motility; both types exhibit a 9 + 2 or 9 + 0 arrangement of microtubule doublets (Mitchison and Valente 2016). Primary cilia, characterized by a 9 + 0 arrangement, are typically solitary and nonmotile. In contrast, motile cilia with a 9 + 2 arrangement are involved in fluid movement across epithelial surfaces, such as the respiratory and female reproductive tracts. Nodal cilia with a 9 + 0 arrangement are located in the embryonic node and play a crucial role in establishing left–right organ asymmetry during human development (Mitchison and Valente 2016). In motile cilia, the inner dynein arms (IDA) and outer dynein arms (ODA) generate the force required for ciliary beating. Two types of ODAs have been identified in mammalian motile cilia. ODA type 1 contains dynein axonemal heavy chain 5 (DNAH5) and dynein axonemal heavy chain 11 (DNAH11), and ODA type 2 contains *DNAH5* and *DNAH9* (Dougherty et al. 2016; Fliegauf et al. 2005).

Variants in the genes encoding the outer dynein arm heavy chains, particularly *DNAH5* and *DNAH11*, account for a substantial fraction of PCD cases. DNAH5 mutations account for 15%–29% of PCD cases in North America and Europe, and 18% of PCD cases in China. These mutations often disrupt ODA assembly, as observed using transmission electron microscopy (TEM), leading to a broad spectrum of clinical phenotypes, including chronic sinusitis, bronchiectasis, neonatal respiratory distress, and situs inversus. Patients with *DNAH5* mutations exhibit high phenotypic heterogeneity, and truncating variants are associated with earlier disease onset and poorer lung function (Dong et al. 2025). *DNAH5* variants causing PCD also presents with genotype heterogeneity, where not only single nucleotide variants (SNVs) and insertion-deletion variants (indels) in *DNAH5* can lead to PCD phenotypes, but also copy number variations (CNVs) in *DNAH5* can also result in PCD phenotypes (Chen et al. 2024). In contrast, DNAH11 variants account for approximately 22% of PCD cases with a normal ciliary ultrastructure. Clinically, *DNAH11-*mutant individuals tend to present with milder respiratory manifestations, including transient neonatal respiratory distress, mild chronic cough, and near-normal nasal nitric oxide (NO) levels. Additionally, approximately 54.5% of patients with biallelic *DNAH11* variants exhibit situs inversus, whereas heterotaxy is observed in approximately 4.5% (Cai et al. 2021; Knowles et al. 2012; Schultz et al. 2020; Shapiro et al. 2020).

Two large-scale genomic studies on PCD identified novel mutations in the *DNAH9* gene, highlighting its role in the pathogenesis of a distinct PCD subtype, primary ciliary dyskinesia 40 (CILD40; OMIM: 618300). In one study, four patients with CILD40 were identified from a cohort of 536 individuals with confirmed or suspected PCD. These patients exhibited situs inversus and upper respiratory tract symptoms, particularly rhinitis, with nasal NO levels within or near the normal range (Fassad et al. 2018). In another study of 548 individuals, five patients from five distinct families were identified as carrying biallelic loss-of-function mutations in the *DNAH9* gene. While all five patients exhibited laterality defects, respiratory symptoms were less prominent and often did not prompt a medical diagnostic workup (Loges et al. 2018). Therefore, CILD40 is characterized by a relatively mild respiratory phenotype, often presenting with situs inversus and normal or low-normal nasal NO levels. *DNAH9* mutations specifically affect the distal region of the cilium, leading to subtle defects in ciliary bending and reduced ciliary clearance. The unique clinical features of individuals with biallelic *DNAH9* mutations have challenged the existing understanding of PCD. Comprehensive characterization of the unique phenotypes associated with *DNAH9* mutations, particularly at the mRNA and protein expression levels, remains underexplored.

In this study, we identified novel compound heterozygous mutations (NM_001372.4:c.308del and NM_001372.4:c.11845G > A) in the *DNAH9* gene associated with CILD40 in a three-generation pedigree. The affected individuals exhibited the typical clinical features of CILD40. Blood transcriptional phenotyping revealed extensive dysregulation of cilium assembly and intraflagellar transport genes, implicating defects in microtubule-based motility pathways in the peripheral blood transcriptome of two affected siblings with biallelic *DNAH9* mutations, but not in their heterozygous family members or one unaffected relative. Plasma proteomic analysis demonstrated significant enrichment of pathways related to RNA splicing, ribosomal function, platelet activation, and complement and coagulation cascades. Moreover, comparisons of plasma proteomic profiles between patients with CILD40 and CILD15 (Kartagener syndrome) revealed differential proteomic patterns in CILD40. These findings provide new insights into the genetic, transcriptional, and proteomic phenotypic heterogeneity underlying CILD40.

## Materials and Methods

### Whole-Exome Sequencing (WES) and Sanger Sequencing

Genomic DNA was extracted from the peripheral blood of all eight family members in a three-generation pedigree. For WES, 50 ng of genomic DNA per sample was enzymatically fragmented into approximately 200 bp fragments, end-repaired, adenylated at the 3′ ends, and ligated to barcoded sequencing adapters. Fragments of approximately 320 bp in size were selected using XP beads and amplified by polymerase chain reaction (PCR) to generate DNA libraries. Exonic regions were captured using the NanoWES kit according to the manufacturer's protocol, followed by elution, PCR amplification, and purification. Library concentrations were determined by quantitative PCR and paired-end (150 bp) sequencing was performed on a NovaSeq 6000 instrument (Illumina, San Diego, CA, USA). Raw reads were processed using CASAVA v1.8.2 for base calling and aligned to the UCSC hg19 reference genome using BWA. PCR duplicates were marked and removed using Picard (http://broadinstitute.github.io/picard/index.html), and variant calling was performed using GATK v4.4.0.0 (https://software.broadinstitute.org/gatk/). Variants were annotated using ANNOVAR (Wang et al. 2010). Rare variants were filtered (population allele frequency < 0.01 in ExAC [r1.2017-07-07], gnomAD_exome [r2.1.1], gnomAD_genome [r3.1], and 1000 Genomes [Phase 3]), and missense variants were assessed using SIFT, PolyPhen-2, MutationTaster, M-CAP, and CADD. Candidate variants identified using WES were subsequently validated in each individual using targeted Sanger sequencing.

### Plasmid Construction

DNAH9 C-terminal cDNAs (nucleotides 1–1200 and 10,845–12,845 of NM_001372.4) were cloned into the pcDNA3.1-FLAG vector (Chongqing Youbao Biotech, Chongqing, China). Site-directed mutagenesis was performed using the KOD-Plus-Mutagenesis Kit (Toyobo, Osaka, Japan) to introduce the patient-derived mutations NM_001372.4:c.308del and NM_001372.4:c.11845G > A (p.A3949T). The following mutation-specific primers were used:

- c.308del-F: GCGCTGGACCTCACTGCCAAGAAAGGTCACTGGGTTATTTTG;
- c.308del-R: TGAGGTCCAGCGCAGCCTCAGCCACCACTTCCTGTCCTTG;
- c.11845G>A-F: CGCTTTTTTCCTTCGCACCGGGCCCGAGCCTCCA; and
- c.11845G>A-R: CGGTGCGAAGGAAAAAAGCGCCTTAGCGCCAGCCAGG.

### Cell Culture and Transfection

HEK-293T cells were maintained in Dulbecco's modified Eagle medium supplemented with 10% fetal bovine serum and 1% penicillin/streptomycin. Cells (~ 60% confluence) were transfected with 4 µg of plasmid DNA using Lipofectamine 3000, following the manufacturer's protocol, and harvested 48 h post-transfection.

### Western Blotting

Cells were lysed in radioimmunoprecipitation assay (RIPA) buffer, and total protein was quantified using a bicinchoninic acid (BCA) assay. Twenty-five micrograms of lysate per lane were resolved using sodium dodecyl sulfate–polyacrylamide gel electrophoresis (SDS–PAGE). The membranes were probed with anti-FLAG (1:1000) and anti-GAPDH (1:1000) antibodies, and the signal was detected using chemiluminescence.

### Bulk RNA Sequencing of the Peripheral Blood Samples

Total RNA was extracted from peripheral blood samples collected from eight individuals in the pedigree using TRIzol Reagent (Life Technologies, Carlsbad, CA, USA). RNA concentration and purity were assessed using a NanoDrop 2000 instrument (Thermo Fisher Scientific, Waltham, MA, USA), and RNA integrity was evaluated using the RNA Nano 6000 Assay Kit on an Agilent Bioanalyzer 2100 system (Agilent Technologies, Santa Clara, CA, USA). One microgram of RNA from each sample was used for library preparation. Sequencing libraries were constructed using the Hieff NGS Ultima Dual-Mode mRNA Library Prep Kit for Illumina (Yeasen Biotechnology, Shanghai, China). MRNA was isolated from total RNA using poly-T oligo–attached magnetic beads, followed by first-strand and second-strand cDNA synthesis. Overhangs were converted to blunt ends through exonuclease/polymerase activity, and after 3′ adenylation, NEBNext adapters with hairpin loops were ligated for library preparation. The library was purified using an AMPure XP system (Beckman Coulter, Beverly, MA, USA). Size-selected, adapter-ligated cDNA was treated with 3 µL of USER Enzyme (NEB, Ipswich, MA, USA) at 37 °C for 15 min, followed by 5 min at 95 °C. PCR amplification was performed using Phusion High-Fidelity DNA Polymerase, universal PCR primers, and an index primer. The PCR products were purified using the AMPure XP system, and library quality was assessed using the Agilent Bioanalyzer 2100 system. Libraries were sequenced on an Illumina NovaSeq platform to generate 150 bp paired-end reads. Reads were aligned to the human genome (hg38) using HISAT2. Alignments were filtered according to standard quality-control criteria, retaining uniquely mapped reads with high mapping quality for downstream analyses. 

### Plasma Protein Enrichment and On-bead Digestion

Plasma proteins were enriched using the n-LAPE/MS™ Kit (ProteinT Biotechnology Co., Ltd.) following the manufacturer’s protocol. Briefly, 80 µL of plasma was mixed with 10 µL of beads and 200 µL of binding buffer, incubated at 37 °C for 10 min with shaking at 1000 rpm, and centrifuged at 12,000 × g for 5 min to remove unbound proteins. Beads were washed three times with 500 µL of Washing Buffer and resuspended in 50 µL of Lysis Buffer (with TCEP and CAA), followed by heating at 95 °C for 10 min, cooling to room temperature, and digested with 2 µL of trypsin buffer for 4 h at 37 °C with agitation. Peptides were precipitated with 950 µL acetonitrile and desalted using the SP3 method on the beads. Eluates (20 µL) were quantified using a NanoDrop instrument prior to liquid chromatography–tandem mass spectrometry (LC–MS/MS; Ma et al. 2023).

### LC–MS/MS and Data-Independent Acquisition (DIA)

Equal amounts of iRT peptides (BioGnosys, Schlieren, Switzerland) were spiked into each run for retention-time calibration. The peptides were injected into a Thermo Scientific UltiMate 3000 RSLCnano UHPLC System (Thermo Fisher) coupled to a Q Exactive HF mass spectrometer. Samples were loaded in 2% ACN/0.1% FA and separated on a 150 µm internal diameter × 25 cm C18 column (1.9 µm, 120 Å; Dr. Maisch GmbH, Ammerbuch, Germany) using a 60-min gradient (A: 2% ACN/0.1% FA; B: 80% ACN/0.1% FA) as follows: 0–5 min, 3–6% B; 6–44 min, 6%–90% B; 45–54 min, 90% B; 55–60 min, re-equilibration; flow rate of 600 nL min⁻^1^. The spray voltage was 2.0 kV (positive mode), and the temperature of the ion-transfer tube was 270 °C. Each cycle began with an MS1 scan (m/z 350–1500) at 60,000 resolution (m/z 200); AGC target = 1e6; and max IT 20 ms, followed by MS2 scans at 30,000 resolution with HCD (NCE 28), AGC target = 1e6, and auto max IT. The fragmentation used 40 variable isolation windows (widths informed by prior DDA surveys), with an MS1 scan preceding each DIA cycle (Ma et al. 2023).

### DIA Data Processing and Identification

Raw DIA files were processed using DIA-NN v1.8.1 (with default settings) against the UniProt human reference proteome (20,365 sequences), with trypsin/P specificity. Identification was performed using neural network-based interference correction in DIA-NN with a false discovery rate (FDR) = 1% at the peptide and protein levels (target-decoy).

### Quantification, Filtering, and Normalization

Samples were retained if they yielded ≥ 3000 identified proteins. Proteins were kept if detected in ≥ 30% of samples. Protein intensities were normalized using the modified FOT normalization (excluding top 20 abundant proteins) (Bader et al. 2023),where the intensity of each protein was normalized as follow: for each sample, (Protein intensity) / (Total protein intensity - Top-20 protein intensity) × 1,000,000. Missing values were imputed as 1 prior to downstream statistical analysis. Reporting followed the HUPO-PSI/MIAPE guidelines, and the datasets were deposited in PRIDE via ProteomeXchange.

### Principal Component Analysis (PCA) and Clustering

To explore the relationships between samples, PCA and hierarchical clustering were applied to the variance-stabilized expression data, which were transformed using the vst function in DESeq2 (version 1.46.0) for visualization purpose. Heatmaps with dendrograms were constructed based on Euclidean distances based hierarchical clustering using function pheatmap and hclust from the R packages pheatmap (version 1.0.12) and stats (version 4.4.1). PCA was calcualted with the prcomp function from stat (version 4.4.1). 

### Differential Expression (DE) Analysis

The filtered reads were counted against Ensembl v76 gene annotations using featureCounts 2.0.8. DE analysis was performed using R (version 4.4.1) and the edgeR package (version 4.4.0). Raw counts were normalized using the trimmed mean of M-values method, followed by the application of the exactTest function in edgeR to detect differentially expressed genes (DEGs) in contrast groups "II-1 vs. Unaffected" and "II-2 vs. Unaffected". *P*-values were corrected for multiple testing using the Benjamini–Hochberg procedure. Genes with a log_2_ fold change |log_2_FC| > 1 and an adjusted *p*-value < 0.01 were considered differentially expressed.

### Enrichment Analysis

Enrichment analysis was conducted separately for upregulated and downregulated DEGs. The clusterProfiler R package (version 4.14.3) was used for gene ID conversion and identification of significantly enriched Gene Ontology (GO) terms and Kyoto Encyclopedia of Genes and Genomes (KEGG) pathways. Terms and pathways with an FDR-adjusted *p*-value < 0.05 were considered significantly overrepresented.

### STRING Enrichment Analysis

Enrichment analysis was performed for genes shared between the DEGs identified in comparisons "II-1 vs. Unaffected" and "II-2 vs. Unaffected" as well as genes in the SCGSv2. In addition, enrichment analysis was conducted for differentially expressed proteins (DEPs) identified in the CILD40 versus Unaffected comparison. Functional enrichment was performed using the STRING database (https://string-db.org). GO terms were identified using STRING's functional enrichment module. Terms were considered significant when they met the following criteria: FDR < 0.05, signal > 0.01, strength > 0.01, and at least two genes/proteins from the input set were present in the enriched term. The top 10 significantly enriched GO terms were visualized using the STRING functional enrichment visualization tool. The signal parameter from STRING was used to represents the ratio between the observed number of genes/proteins associated with a given functional term and the expected number based on the background distribution. To reduce redundancy among enriched terms, term similarity was calculated using the STRING functional enrichment visualization module based on the overlap of genes assigned to each term. Terms with a similarity score greater than 0.8 were grouped into a single cluster, and one representative term was displayed for visualization.

### DEP Analysis

We performed sample screening and decided to remove sample III-2 and the father of a patient with CILD15 because of insufficient protein detection during quality control. Subsequently, raw abundance data were normalized using the modified FOT method. In cases where missing values were present, they were replaced with 1 prior to data analysis. This rigorous approach ensured data quality and robust analysis. PCA and clustering analyses were performed, as previously described, using the modified FOT normalized data. Given the high similarity observed between samples II-1 and II-2 in PCA and clustering, these samples were grouped into the CILD40 contrast group for subsequent analysis. Differential protein expression analysis was performed using the exactTest function in the edgeR package. Although edgeR was originally developed for RNA-seq count data, recent benchmark studies have demonstrated that RNA-seq-based statistical methods, including edgeR, can be effectively applied to quantitative proteomics data after appropriate preprocessing and achieve performance comparable to or exceeding several proteomics-specific methods (Lin et al. 2022). The exactTest procedure was selected because our study involved limited replications, for which it provides a suitable statistical framework for two-group differential expression analysis. *P*-values were corrected using the same procedure as for the RNA-seq DE analysis. Proteins with a |log_2_ FC| > 1 and an adjusted *p*-value < 0.01 were considered differentially expressed.

### Protein–Protein Interaction (PPI) Network Construction

The top 50 percent of DEPs, ranked by |log_2_FC|, were selected to construct the PPI network using the STRING database. The resulting PPI network was analyzed using Cytoscape (version 3.10.3). Nodes with a degree ≥ 5 were retained for further analysis. Hub proteins were identified using the Maximal Clique Centrality (MCC) ranking method implemented in the cytoHubba plugin. The GLay community clustering algorithm was applied to hub proteins and their first neighbors.

### Protein Structure Prediction

The protein structures of the wild-type (WT) and mutant DNAH9 were predicted using the trRosetta web server. The WT *DNAH9* sequence (UniProt accession number: Q9NYC9) was retrieved from the UniProt database. For the NM_001372.4:c.11845G > A (p.A3949T) mutation, the original sequence was truncated to a 1000-amino-acid segment centered on the mutation site. The frameshift and missense mutations (NM_001372.4:c.308delT:p.F103Sfs31, NM_001372.4:c.308dupT:p.Leu104Profs45 and NM_001372.4:c.11666C > G:p.Ser3889*) and the canonical transcript sequence for *DNAH9* were retrieved from Ensembl, and a truncated protein sequence was generated by translating the mutated sequence using Biopython (version 1.84). The predicted protein structure and hydrogen bonds were analyzed and visualized using PyMOL (https://github.com/schrodinger/pymol-open-source).

## Results

### Clinical Features and Molecular Genetics of a Three-Generation Pedigree with Novel Compound Heterozygous Mutations in *DNAH9*

Situs ambiguus is characterized by abnormal visceral situs and associated malformations, and is often linked to left atrial isomerism (Montero-Macías et al. 2023). The imaging findings previously documented in this proband included the absence of the retrohepatic segment of the inferior vena cava, direct hepatic vein drainage into the right atrium, congenital pancreatic hypoplasia with duodenal stenosis, polysplenia, and intrahepatic bile duct dilation (Chen et al. 2025; Kong et al. 2025). These findings collectively supported the initial diagnosis of situs ambiguous. After inquiring about the family history, it was found that only the proband's older sister exhibited mirror-image dextrocardia, which is a characteristic feature of situs inversus and is often associated with PCD (Lucas et al. 2014). Both sisters reported no respiratory complaints, such as chronic cough or nasal congestion, and neither experienced otitis media or ectopic pregnancies. Clinical evaluation revealed no evidence of rhinosinusitis, recurrent respiratory infections, or bronchiectasis, which are common manifestations of PCD. No such clinical manifestations were observed in other family members within the three-generation pedigree (Table 1).

**Table 1:** PCD subtypes from OMIM database

Phenotype	Gene/Locus	Phenotype-MIM number	Gene/Locus MIM number	Deficiency	PMID
Ciliary dyskinesia, primary, 1	*DNAI1*	244400	604366	ODA	25610612
Ciliary dyskinesia, primary, 2	*DNAAF3*	606763	614566	IDA + ODA	25610612
Ciliary dyskinesia, primary, 3	*DNAH5*	608644	603335	ODA	25610612
Ciliary dyskinesia, primary, 4	*CILD4*	608646	608646	n.a	14985390
Ciliary dyskinesia, primary, 5	*HYDIN*	608647	610812	CP + RS	29800551
Ciliary dyskinesia, primary, 6	*NME8*	610852	607421	ODA	29800551
Ciliary dyskinesia, primary, 7	*DNAH11*	611884	603339	ODA	29800551
Ciliary dyskinesia, primary, 8	*CILD8*	612274	612274	n.a	16397065
Ciliary dyskinesia, primary, 9	*DNAI2*	612444	605483	ODA	29800551
Ciliary dyskinesia, primary, 10	*DNAAF2*	612518	612517	IDA + ODA	29800551
Ciliary dyskinesia, primary, 11	*RSPH4A*	612649	612647	RS	29800551
Ciliary dyskinesia, primary, 12	*RSPH9*	612650	612648	RS	29800551
Ciliary dyskinesia, primary, 13	*DNAAF1*	613193	613190	IDA + ODA	29800551
Ciliary dyskinesia, primary, 14	*CCDC39*	613807	613798	IDA	29800551
Ciliary dyskinesia, primary, 15	*CCDC40*	613808	613799	IDA	29800551
Ciliary dyskinesia, primary, 16	*DNAL1*	614017	610062	ODA	29800551
Ciliary dyskinesia, primary, 17	*DNAAF19*	614679	614677	IDA + ODA	22581229
Ciliary dyskinesia, primary, 18	*DNAAF5*	614874	614864	IDA + ODA	29800551
Ciliary dyskinesia, primary, 19	*DNAAF11*	614935	614930	IDA + ODA	23122589
Ciliary dyskinesia, primary, 20	*ODAD1*	615067	615038	ODA	23261303
Ciliary dyskinesia, primary, 21	*DRC1*	615294	615288	DRC/NL	23354437
Ciliary dyskinesia, primary, 22	*ZMYND10*	615444	607070	IDA + ODA	29800551
Ciliary dyskinesia, primary, 23	*ODAD2*	615451	615408	ODA	3888613;3738828
Ciliary dyskinesia, primary, 24	*RSPH1*	615481	609314	CP + RS	29800551
Ciliary dyskinesia, primary, 25	*DNAAF4*	615482	608706	IDA + ODA	23872636
Ciliary dyskinesia, primary, 26	*CFAP298*	615500	615494	IDA + ODA	24094744
Ciliary dyskinesia, primary, 27	*CCDC65*	615504	611088	NL	25610612
Ciliary dyskinesia, primary, 28	*SPAG1*	615505	603395	IDA + ODA	29800551
Ciliary dyskinesia, primary, 29	*CCNO*	615872	607752	n.a	29800551
Ciliary dyskinesia, primary, 30	*ODAD2*	616037	615956	ODA	24203976;23849778
Ciliary dyskinesia, primary, 32	*RSPH3*	616481	615876	CP + RS	29800551
Ciliary dyskinesia, primary, 33	*GAS8*	616726	605178	DRC	25610612
Ciliary dyskinesia, primary, 34	*DNAJB13*	617091	610263	CP + RS	29800551
Ciliary dyskinesia, primary, 35	*ODAD4*	617092	617095	ODA	27486780
Ciliary dyskinesia, primary, 36	*DNAAF6*	300991	300933	IDA + ODA	28041644;24421334
Ciliary dyskinesia, primary, 37	*DNAH1*	617577	603332	IDA	29800551
Ciliary dyskinesia, primary, 38	*CFAP300*	618063	618058	IDA + ODA	29727692;29727693
Ciliary dyskinesia, primary, 39	*LRRC56*	618254	618227	ODA	30388400
Ciliary dyskinesia, primary, 40	*DNAH9*	618300	603330	ODA	30471717
Ciliary dyskinesia, primary, 41	*GAS2L2*	618449	611398	Ciliary basal	30665704
Ciliary dyskinesia, primary, 42	*MCIDAS*	618695	614086	n.a	29800551
Ciliary dyskinesia, primary, 43	*FOXJ1*	618699	602291	n.a	31630787
Ciliary dyskinesia, primary, 44	*NEK10*	618781	618726	n.a	31959991
Ciliary dyskinesia, primary, 45	*TTC12*	618801	610732	IDA + ODA	31978331
Ciliary dyskinesia, primary, 46	*STK36*	619436	607652	CP + RS	28543983
Ciliary dyskinesia, primary, 47	*TP73*	619466	601990	n.a	34077761
Ciliary dyskinesia, primary, 48	*NME5*	620032	603575	RS	32185794
Ciliary dyskinesia, primary, 49	*CFAP74*	620197	620187	DMT	32555313
Ciliary dyskinesia, primary, 50	*DNAH7*	620356	610061	IDA	11877439
Ciliary dyskinesia, primary, 51	*BRWD1*	620438	617824	IDA + ODA	33389130
Ciliary dyskinesia, primary, 52	*DAW1*	620570	620279	ODA	18852297
Ciliary dyskinesia, primary, 53	*CLXN*	620642	619564	ODA	36727596

n.a: no relevant to ciliary structure or function; ODA: Outer dynein arm; IDA: Inner dynein arm; CP: Central pair microtubules;RS: radial spoke; DRC: dynein regulatory complex; NL: nexin link; DMT: doublet microtubule

The diagnosis and treatment of peripancreatic and retroperitoneal space–occupying lesions in the proband (II-2) are summarized below. Briefly, the proband (II-2, 38 years old), who had choledocholithiasis and underwent laparoscopic cholecystectomy and choledocholithotomy 2 years before presentation, attended the Hepatobiliary Surgery Clinic of Beijing Tsinghua Changgung Hospital in January 2023 with progressive obstructive jaundice, severe abdominal distension, and weight loss. Imaging revealed intrahepatic duct dilatation, hepatic portal bile duct stricture, and fibrotic retroperitoneal/peripancreatic masses extending to the liver ligaments (Kong et al. 2025). Markedly elevated CA 19–9 (1,334 U/L) and inflammatory markers (CRP 64.52 mg/L, ESR 77 mm/h) levels were noted. Biopsy of the peripancreatic mass revealed a poorly differentiated adenocarcinoma, which was confirmed by multidisciplinary molecular profiling to establish the final diagnosis of retroperitoneal fibrosis secondary to metastatic pancreatic cancer. Palliative chemotherapy was not administered because of the patient's poor tolerance. In late March, the patient was discharged in a stable condition, receiving basic total parenteral nutrition treatment, and eventually died 2 months later.

**Fig. 1:**
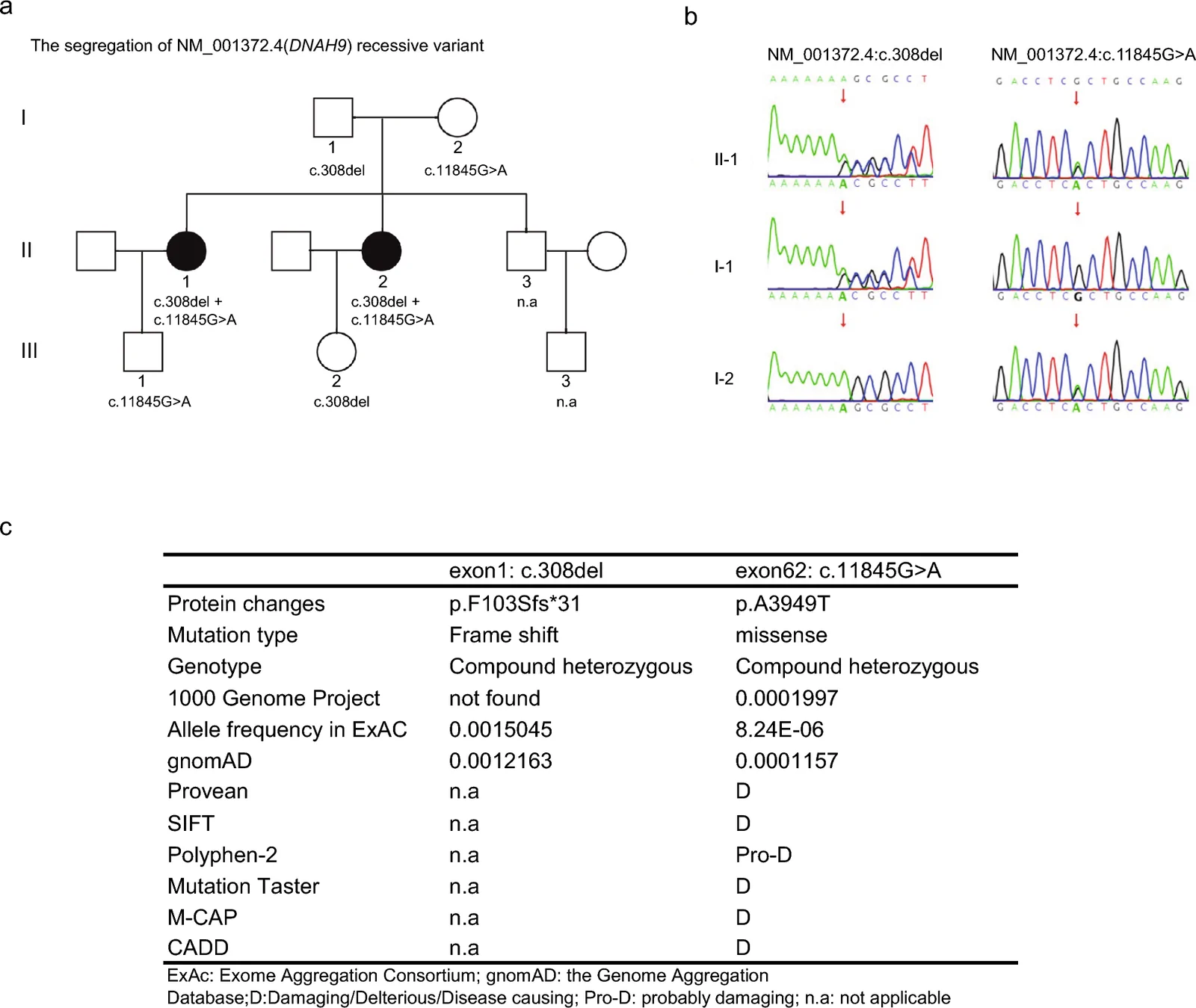
Screening of pathogenic mutation sites in the pedigree. **a** Pedigrees of the proband's family indicating the affected individuals (filled in black) and segregation of *DNAH9* recessive variants. **b** Sanger sequencing of *DNAH9* variants in Patient 1 and the unaffected parents. **c** Variant analysis of the two *DNAH9* mutations

To investigate the genetic underpinnings of laterality defects and other clinical manifestations, WES was performed for eight members of the three-generation pedigree, including the proband's parents (first generation, I-1/2), the proband (second generation, II-2), the proband's sister (second generation, II-1), the proband's brother (second generation, II-3), and three children of each of the second-generation members (third generation, III-1/2/3) (Fig. 1a). The sequencing results showed that the proband and her older sister had novel compound heterozygous mutations in *DNAH9*, including NM_001372.4:c.308del (p.F103Sfs*31) and NM_001372.4:c.11845G > A (p.A3949T). These mutations were inherited from the proband’s father and mother, as confirmed by Sanger sequencing (Fig. 1b). The NM_001372.4:c.308del frameshift mutation is rare, whereas the NM_001372.4:c.11845G > A missense mutation is extremely rare in public databases, including the ExAC Browser (0.0015045), gnomAD (0.0012163), and the 1000 Genomes Project (not found). The prediction results from SIFT, PolyPhen-2, MutationTaster, M-CAP, and CADD suggested that the NM_001372.4:c.11845G > A mutation was probably damaging (Fig. 1c).

### Novel Compound Heterozygous Mutations in *DNAH9* Disrupted Protein Function and Determined the Clinical Features of CILD40

**Fig. 2:**
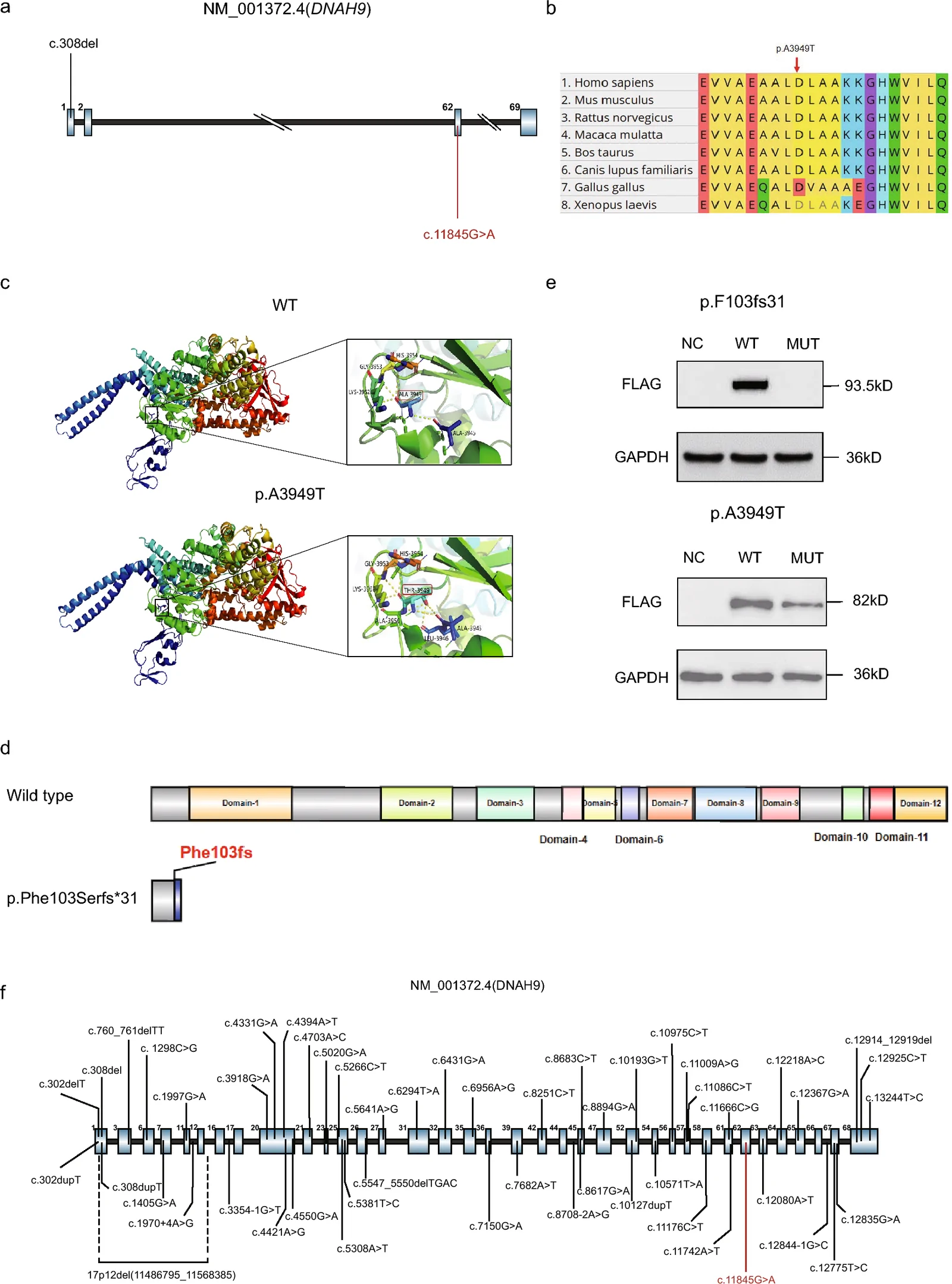
Novel compound heterozygous mutations (NM_001372.4:c.11845G > A and NM_001372.4:c.308del) in *DNAH9*. **a** Genomic location of the two *DNAH9* mutation sites. **b** Multiple sequence alignment of the mutated amino acid site in *DNAH9* proteins of different species. Red arrows indicate the position of the variant p.A3949T. **c** The structural alteration of the p.A3949T protein variant. The A3949 (WT) and T3949 (mutant) residues are highlighted by the red box in the enlarged image. In the wild type, A3949 forms hydrogen bonds (yellow dotted lines) with L3952, G3953, H3954, and ALA3945 residues, respecticely. In the mutant, T3949 forms new hydrogen bonds with the adjacent A3950 and L3946 residues. **d** Protein domain presentation of the truncated protein caused by the frameshift p.Phe103Serfs*31 mutation. **e** Western blotting analysis of FLAG-tagged DNAH9 expression in HEK-293T cells. HEK-293T cells were transfected with an empty vector control (NC), wild-type DNAH9–FLAG (WT), or mutant DNAH9-FLAG constructs carrying either the c.308del frameshift variant (p.F103Sfs31) or the c.11845G > A missense substitution (p.A3949T). GAPDH served as a loading control. **f** Schematic presentation of the *DNAH9* genomic structure. The positions of the *DNAH9* mutations identified from previous studies are indicated

To evaluate the impact of these mutations on protein functionality, we conducted a comprehensive analysis of protein structure and function. NM_001372.4:c.308del (p.F103Sfs*31) and NM_001372.4:c.11845G > A (p.A3949T) were located in exon 1 and exon 62, respectively (Fig. 2a). Bioinformatic analysis showed that the amino acid at position 3,949 was highly conserved among mammalian species (Fig. 2b). Furthermore, structural analysis of the p.A3949T variant revealed that the mutation disrupted hydrogen bond formation (Fig. 2c), potentially affecting the stability of the secondary structure of *DNAH9*. Meanwhile, the frameshift mutation at nucleotide 308 led to premature termination after the translation of an additional 31 amino acids, beginning at residue 103 (Fig. 2d). This resulted in a truncated *DNAH9* protein and subsequent loss of function (Fig. S1a). To validate the effects of these variants on DNAH9 stability, FLAG-tagged WT and mutant (p.F103Sfs31 and p.A3949T) constructs were expressed in HEK293T cells, and cell lysates were analyzed by western blotting. The p.F103Sfs31 mutant failed to yield a detectable ∼93.5 kDa band, indicating that the truncated protein was unstable or rapidly degraded. In contrast, the p.A3949T variant produced a discrete ∼82 kDa band of substantially lower intensity than the WT band, consistent with the partial destabilization of the missense protein (Fig. 2e). These results demonstrated that the F103Sfs*31 frameshift abolished stable DNAH9 expression, whereas the A3949T missense mutation compromised protein integrity.

In the genes responsible for the 53 identified subtypes of PCD, 52.83% (28/53) of the pathogenic variants were associated with ODA dysfunction (Table 2). We then conducted a comparative genotype–phenotype analysis of previously reported *DNAH9* mutations (Al‐Korashy et al. 2024; Chen et al. 2022; Fassad et al. 2018; Feng et al. 2022; Isa et al. 2022; Justin Margret et al. 2024; Loges et al. 2018; Reuter et al. 2020; Rojnueangnit et al. 2022; Takeuchi et al. 2021; Tang et al. 2021; Tate 2021; Yan et al. 2024; Zheng et al. 2022) (Table 3). *DNAH9* comprised 69 exons, with compound heterozygous mutations identified in 25 exons and homozygous mutations in eight exons (Fig. 2f). The highest frequency of mutations was observed in exon 20, highlighting its critical role in the pathogenesis of *DNAH9*-associated disorders. Mutations affecting *DNAH9* have been previously identified in patients carrying the 308dupT and c.11666C > G mutations (Chen et al. 2022). Both mutations led to a truncated *DNAH9* protein, resulting in loss of function (Fig. S1b, c). Among the 31 patients with CILD40, 23 (74.19%) carried compound heterozygous mutations, seven (22.58%) had homozygous mutations, and only one (3.23%) harbored a single heterozygous mutation in *DNAH9*. The patient with a single heterozygous mutation also carried a heterozygous mutation (c.121G > A) in *RSPH1*, which is essential for ciliary function. Typical clinical features of CILD40 include mild respiratory symptoms, laterality defects, and cardiac-related malfunctions. Among the patients with *DNAH9* mutations with available phenotypic data, 72.41% (21/29) had laterality defects; 44.44% (12/27) exhibited respiratory symptoms, including wet cough, rhinosinusitis, and bronchiectasis; and 36.67% (11/30) presented with cardiac-related malfunctions. Among the patients with compound heterozygous mutations, 65.21% (15/23) exhibited laterality defects, 34.78% (8/23) experienced respiratory symptoms, 21.74% (5/23) were infertile, and 43.48% (10/23) displayed cardiac-related malfunctions, indicating considerable phenotypic heterogeneity associated with compound heterozygous mutations in *DNAH9* (Table 3). These findings strongly suggested that compound heterozygous mutations in *DNAH9* were the predominant variant types that significantly affect protein integrity and function, and were key determinants of clinical phenotypes.

**Table 2:** Summary of all *DNAH9* mutations and major clinical manifestations from previous studies

Patient ID/reference	Genomic mutation	Genomic location	Protein change	Mutation type	Genotype	Respiratory	Laterality defect	Infertile	Cardiac defect
Fassad et al. (2018)									
1.II.1	c.12367G > A	exon65	p.Asp4123Asn	Missense mutation	Homozygous	Yes	Yes	n.a	Yes
2.II.2	c.8708-2A > G c.10193G > T	exon 44—exon45(intron) exon52	p.Glu2904Aspfs*53 p.Arg3398Leu	Frameshift mutation Missense mutation	Compound heterozygous	Yes	Yes	n.a	No
3.II.1	c.5641A > G	exon27	p.Lys1881Glu	Missense mutation	Homozygous	Yes	Yes	Yes	No
3.II.1	c.8894G > A	exon47	p.Arg2965His	Missense mutation	Homozygous	Yes	Yes	Yes	No
Loges et al. (2018)									
OP-2905 II1	c.1970 + 4 A > G c.3354-1G > T	exon11—exon 12 (intron) exon 16—exon17(intron)	intronic_splice_site intronic_splice_site	Splicing mutation Splicing mutation	Compound heterozygous	Yes	Yes	n.a	No
MD II1	c.1997G > A c.5020G > A	exon11 exon23	p.Trp666* p.Gly1674Arg	Nonsense mutation Missense mutation	Compound heterozygous	Yes	Yes	n.a	No
MS-SI46 II1	c.10127dupT	exon52	p.Leu3376Phefs*57	Frameshift mutation	Homozygous	Yes	Yes	n.a	No
Reuter et al. (2020)									
HSC_087	c.4421A > G c.13244 T > C	exon20 exon68	p.Asp1474Gly p.Ile4415Thr	Missense mutation Missense mutation	Compound heterozygous	Yes	Yes	n.a	Yes
Tang et al. (2021)									
F1 II-1	c.302dupT c.6956A > G	exon1 exon35	p.Leu101fs*47 p.Asp2319Gly	Frameshift mutation Missense mutation	Compound heterozygous	No	No	Yes	No
F2 II-1	c.6294 T > A c.10571 T > A	exon31 exon54	p.Phe2098Leu p.Leu3524Gln	Missense mutation Missense mutation	Compound heterozygous	No	No	Yes	No
Takeuchi et al. (2021)									
unamed_2	c. 1298 C > G c.5547_5550delTGAC	exon 6 exon26	p.Ser433Cys p.Asp1850fs	Missense mutation Frameshift mutation	Compound heterozygous	Yes	No	n.a	No
Tate (2021)									
unamed_fetus_2	c.12775 T > C	exon67	p.C4259R	Missense mutation	Heterozygous	n.a	Yes	n.a	No
Chen et al. (2022)									
F1-II	c.5308A > T c.5381 T > C	exon25 exon25	p.K1770Ter p.I1794T	Nonsense mutation Missense mutation	Compound heterozygous	No	Yes	n.a	Yes
F2-II	c.760_761delTT c.11176C > T	exon3 exon58	p.Phe254Leufs*6 p.R3726W	Frameshift mutation Missense mutation	Compound heterozygous	No	No	n.a	No
F3-II	c.4703A > C c.11009A > G	exon21 exon57	p.N1568T p.E3670G	Missense mutation Missense mutation	Compound heterozygous	No	Yes	n.a	Yes
F4-II	c.12844-1G > C c.12080A > T	exon 66—exon67(intron) exon63	intronic_splice_site p.H4027L	Splicing mutation Missense mutation	Compound heterozygous	No	Yes	n.a	Yes
F5-II	c.302delT c.5381 T > C	exon1 exon25	p.F103SfsTer31 p.I1794T	Frameshift mutation Missense mutation	Compound heterozygous	No	Yes	n.a	Yes
F6-II	c.302dupT c.11176C > T	exon1 exon58	p.L104PfsTer45 p.R3726W	Frameshift mutation Missense mutation	Compound heterozygous	No	No	n.a	Yes
Individual-7	c.12914_12919del c.7682A > T	exon68 exon39	p.4305_4307delSWA p.H2561L	Deletion mutation Missense mutation	Compound heterozygous	No	Yes	Yes	No
Individual-8	c.4394A > T c.8683C > T	exon20 exon45	p.D1465V p.L2895F	Missense mutation Missense mutation	Compound heterozygous	No	Yes	n.a	Yes
unamed_fetus_1	c.3918G > A c.7150G > A	exon20 exon36	p.Trp1306* p.Gly2384Arg	Nonsense mutation Missense mutation	Compound heterozygous	No	Yes	n.a	Yes
Zheng et al. (2022)									
unamed_1	c.6431G > A c.12835G > A	exon32 exon67	p.R2144Q p.G4279S	Missense mutation Missense mutation	Compound heterozygous	Yes	No	Yes	No
Tate (2021)									
unamed_fetus_2	c.12775 T > C	exon67	p.C4259R	Missense mutation	Heterozygous	n.a	Yes	n.a	No
Zhang et al. (2022)									
unamed_fetus_3	c.10975C > T g.11486795_11568385del	exon56 exon1—exon 15 (del)	p.Q3659* not applicable	Nonsense mutation Deletion mutation	Compound heterozygous	n.a	Yes	n.a	Yes
Feng et al. (2022)									
unamed_3	c.760_761delTT c.4331G > A	exon3 exon20	p.Phe254Leufs*6 p.Trp1444*	Frameshift mutation Nonsense mutation	Compound heterozygous	Yes	Yes	n.a	No
Isa et al. (2022)									
unamed_4	c.11086C > T c.7150G > A	exon57	p.His3696Tyr	Missense mutation	Compound heterozygous	Yes	Yes	n.a	No
Rojnueangnit et al. (2022)									
patient 7	c.5266C > T	exon25	p.Gln1756Ter	Nonsense mutation	Compound heterozygous	n.a	n.a	n.a	No
Justin Margret et al. (2024)									
DIS-8	c.1405G > A c.4550G > A	exon7 exon20	p.Gly469Ser p.Arg1517Gln	Missense mutation Missense mutation	Compound heterozygous	n.a	n.a	Yes	No
Yan et al. (2024)									
AS001	c.12218A > C	exon64	p.N4073T	Missense mutation	Homozygous	No	No	n.a	No
AS009	c.8617G > A c.11742A > T	exon45 exon61	p.V2873M p.E3914D	Missense mutation Missense mutation	Compound heterozygous	No	No	n.a	No
Al-Korashy et al. (2024)									
F15-20	c.12925C > T	exon68	p.Arg4309*	Nonsense mutation	Homozygous	No	Yes	n.a	No

n.a: not applicable

**Table 3:** Clinical manifestations in a three-generation pedigree

	II-1	II-2	I-1	I-2	II-3	III-1	III-2	III-3
Respiratory phenotype	N	N	N	N	N	N	N	N
Rhinosinusitis	N	N	N	N	N	N	N	N
Chronic cough	N	N	N	N	N	N	N	N
Bronchiectasis	N	N	N	N	N	N	N	N
Otitis media	N	N	N	N	N	N	N	N
Situs Inversusim	N	Y	N	N	N	N	N	N
Ectopic pregnency	N	N	N	N	N	N	N	N
Left isomerism	Y	N	N	N	N	N	N	N

Y: Yes; N: No

### Altered Peripheral Blood Gene Signature in Patients with CILD40 Carrying Novel Compound Heterozygous Mutations in *DNAH9*

**Fig. 3:**
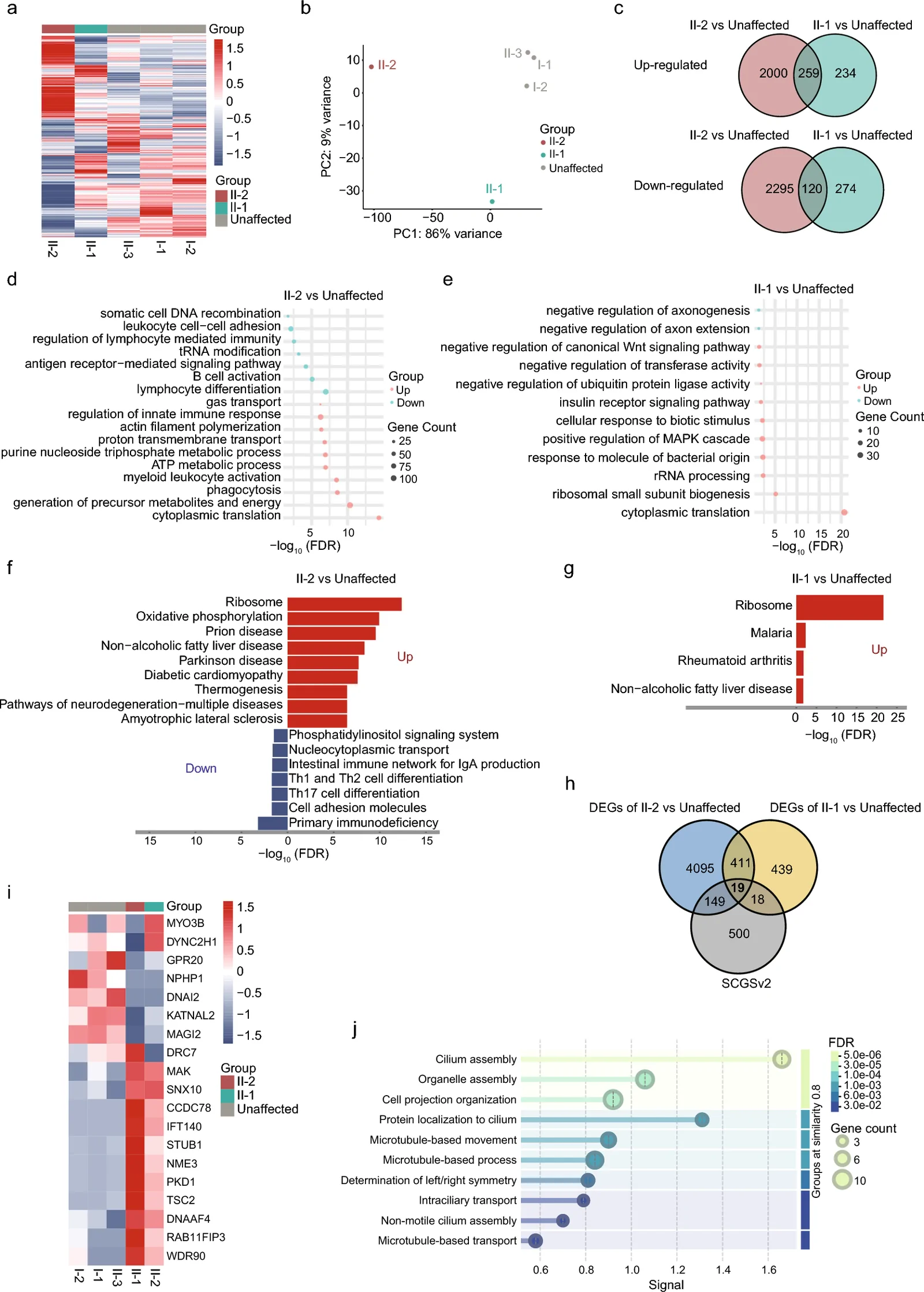
Altered peripheral blood gene expression profiles in CILD40. **a** Heatmap showing the normalized RNA expression patterns in adult samples. **b** Principal component analysis (PCA) plots of all pedigree samples. **c** Venn diagram showing differentially expressed genes in II-1 and II-2. **d–e** Bubble plot of the top 10 significantly enriched Gene Ontology (GO) terms (bubble plot only; regulation status is indicated by color) of two patients with primary ciliary dyskinesia (PCD) (false discovery rate [FDR]-adjusted p-value < 0.05). **f–g** The bar plot of the top 10 significantly enriched Kyoto Encyclopedia of Genes and Genomes (KEGG) pathways of two patients with PCD (FDR < 0.05). Regulation status is indicated by the color and orientation of the bar. **h** Venn diagram illustrating the overlap among differentially expressed genes (DEGs) in II-1 vs. unaffected, II-2 vs. unaffected, and the SysCilia Gold Standard v2 (SCGSv2) gene sets. **i** Heat map of the 19 overlapping DEGs. **j** Bubble plot of GO biological process enrichment for the 19 overlapping DEGs, with bubble size proportional to gene count and color denoting –log₁₀(FDR)

To investigate the alterations in gene expression associated with novel compound heterozygous mutations in *DNAH9*, bulk RNA sequencing was performed on peripheral blood samples collected from a three-generation pedigree. Clustering analysis and PCA revealed heterogeneity between the CILD40-only sample (proband's sister, II-1) and the CILD40 with tumor sample (proband, II-2) (Fig. 3a, b). DE analysis identified 4674 differentially expressed genes (DEGs) in II-2, including 2259 upregulated and 2415 downregulated genes. In II-1, 887 DEGs were detected, including 493 upregulated and 394 downregulated genes (Table S1). Among these, 259 (10.4%) upregulated genes and 120 (4.5%) downregulated genes overlapped between II-1 and II-2, suggesting a shared transcriptional dysregulation related to CILD40 (Fig. 3c).

To assess the biological impact of these alterations, we performed GO enrichment analysis. In II-2, downregulated DEGs were significantly enriched in pathways related to lymphocyte differentiation (GO:0030098; *q*-value = 1.04E-07), B cell activation (GO:0042113; *q*-value = 8.19E-06), leukocyte adhesion (GO:0007159; *q*-value = 7.0E-03), and the regulation of lymphocyte-mediated immunity (GO:0002706; *q*-value: 3.0E-03), whereas upregulated DEGs were predominantly associated with metabolic processes, innate immune response regulation (GO:0045088; *q*-value = 5.62E-07), and proton transmembrane transport (GO:1902600; *q*-value = 1.51E-07) (Fig. 3d, Table S1). In II-1, downregulated DEGs were notably enriched in the negative regulation of axonogenesis (GO:0050771; *q*-value = 4.6E-02), whereas upregulated DEGs were mainly associated with the MAPK cascade (GO:0043410; *q*-value = 7.0E-03), rRNA processing (GO:0006364; *q*-value = 5.0E-03), and responses to bacterial molecules (GO:0002237; *q*-value = 5.0E-03) (Fig. 3e, Table S1). KEGG analysis revealed that in II-2, upregulated pathways were significantly enriched in processes related to ribosome function (hsa03010; *q*-value = 3.59E-13), oxidative phosphorylation (hsa00190; *q*-value = 1.01E-10), and various diseases, while downregulated pathways were primarily associated with primary immunodeficiency (hsa05340; *q*-value = 5.68E-04), cell adhesion molecules (hsa04514; *q*-value = 1.8E-02), and Th17 cell differentiation (hsa04659; *q*-value = 1.8E-02 ) (Fig. 3f). In II-1, the upregulated pathways were significantly enriched in processes related to ribosomal function (hsa03010; *q*-value = 2.07E-22) (Fig. 3g, Table S1).

To validate the ciliary relevance of transcriptomic dysregulation in CILD40, we cross-referenced the overlapping DEGs of the proband (II-2) and her sister (II-1) against the SYSCILIA Gold Standard v2 (SCGSv2), a manually curated set of 686 expert-annotated ciliary proteins (Vasquez et al. 2021). In II-2, 168 DEGs overlapped with SCGSv2, whereas the sister (II-1) contributed to 37 ciliary DEGs. Nineteen genes were common to both individuals (Fig. 3h, i). These shared genes were significantly enriched in core and related ciliary processes, including Cilium assembly (GO:0060271; FDR = 5.71E-06), Intraflagellar transport (GO:0042073; FDR = 1.62E-02), Microtubule-based movement (GO:0007018; FDR = 2.2E-03), and Protein localization to cilium (GO:0061512; FDR = 8.4E-04) (Fig. 3j, Table S1). This convergent dysregulation of evolutionarily conserved ciliary-related biological processes provided compelling evidence that the observed transcriptional signatures were directly due to loss-of-function of *DNAH9*, with potential downstream effects on immune signaling, ribosome biogenesis, cell motility, and broader disease susceptibility.

### Distinct Plasma Protein Signatures Associated with Novel Compound Heterozygous Mutations in *DNAH9*

**Fig. 4:**
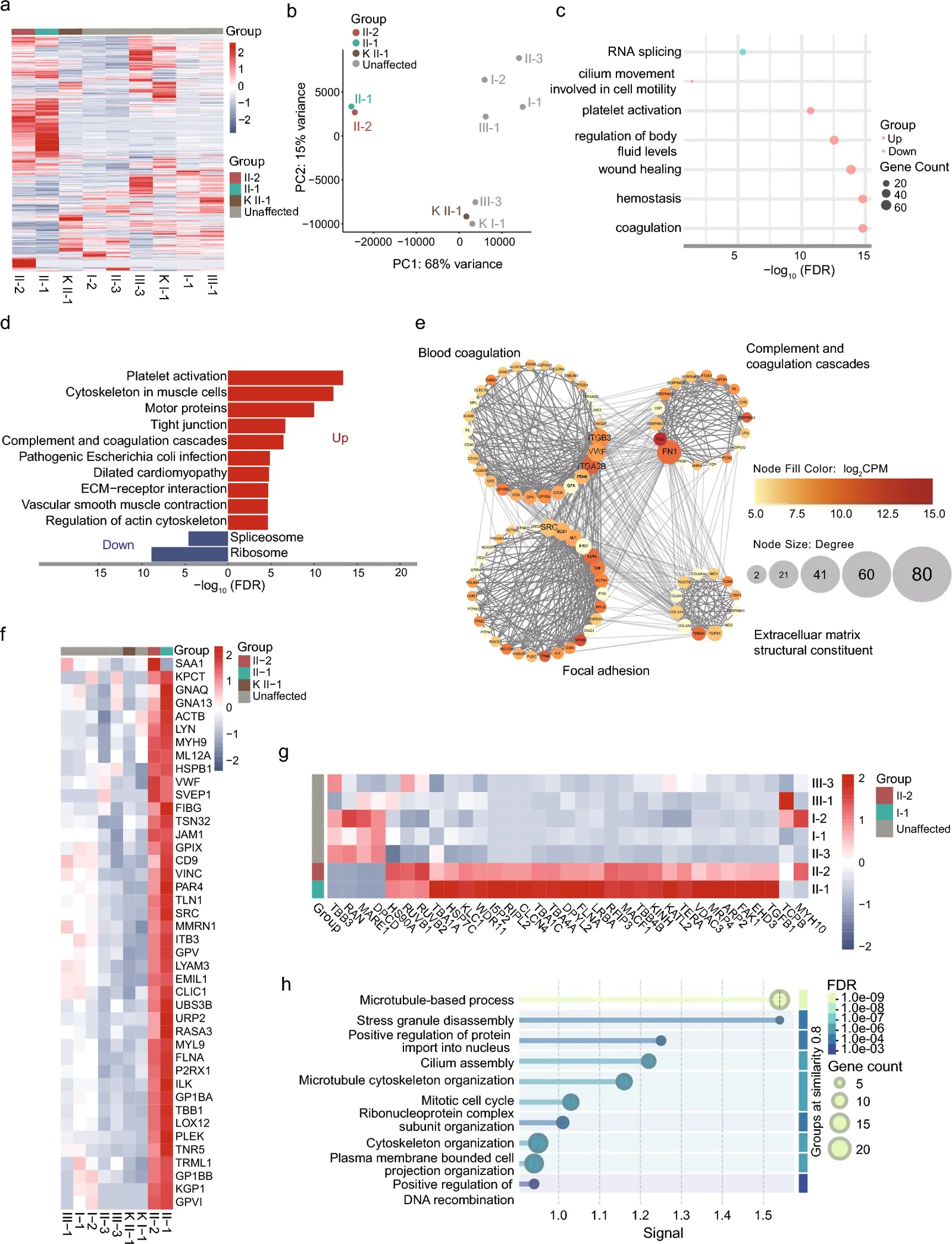
Plasma protein signatures in CILD40. **a** Heatmap showing the normalized protein expression patterns among all samples, including a CILD15 patient and his healthy mother. **b** PCA plot of all the samples. **c** The bubble plot presents the 10 most significantly enriched GO terms in patients with CILD40. **d** The bar plot shows the 10 most enriched KEGG pathways in CILD40 patients. **e** The GLay community clustering was performed in the protein–protein interaction (PPI) network of hub proteins and their first neighbors. The lightened edges indicate inter-community interactions. **f** Heatmap of 42 differentially expressed proteins (DEPs) shared among three coagulation-related GO terms. **g** Heatmap of the 31 DEPs shared between plasma DEPs and SCGSv2. **h** Bubble plot of GO biological process enrichment for 31 overlapping DEPs

The plasma proteome is a vital resource for capturing characteristic phenotypes and identifying potential diagnostic biomarkers (Niu et al. 2022). To investigate the plasma proteomic alterations linked to CILD40, we first compared the protein expression changes associated with biallelic mutations in *DNAH9* between heterozygous family members and an unaffected relative. The results showed that samples with biallelic mutations in *DNAH9* grouped together, suggesting a similar blood protein expression pattern in CILD40 (Fig. 4a, b). DE analysis identified 876 DEPs in the CILD40 group, including 668 upregulated and 208 downregulated proteins (Table S2). To explore the potential biological effects of specific alterations in CILD40, we performed GO enrichment analysis. The results showed that the downregulated DEPs were significantly enriched in pathways related to RNA splicing (GO:0008380; *q*-value = 2..8E-5). The upregulated DEPs were mainly associated with coagulation (hsa03040; *q*-value = 1.31E-15), hemostasis (GO:0007599; *q*-value = 1.31E-15), platelet activation (GO:0030168; *q*-value = 1.63E-11), and cilium movement involved in cell motility (GO:0060294; *q*-value = 3.1E-2) (Fig. 4c, Table S2). KEGG analysis revealed that the upregulated pathways in CILD40 were significantly enriched in processes related to platelet activation (hsa04611; *q*-value = 3.33E-14), cytoskeleton in muscle cells (hsa04820; *q*-value = 4.47E-13), motor protein (hsa04814; *q*-value = 7.99E-11), tight junction (hsa04530; *q*-value = 1.76E-7), and complement and coagulation cascades (hsa04610; *q*-value = 2.85E-7). The pathways downregulated in CILD40 were primarily associated with spliceosome (hsa03040; *q*-value = 2.58E-5) and ribosome (hsa03010; *q*-value = 1.28E-9) (Fig. 4d, Table S2). The key proteins identified in PPI networks, including FN1 (log_2_ counts per million [CPM] = 11.08, degree = 72), ITGB3 (log_2_CPM = 9.93, degree = 45), VWF (log_2_CPM = 8.70, degree = 44), SRC (log_2_CPM = 5.96, degree = 42) and ITGA2B (log_2_CPM = 11.47, degree = 42), were primarily involved in blood coagulation (GO:0007596; FDR = 2.84E-15), complement and coagulation cascades (hsa04610; FDR = 1.94E-12), focal adhesion (GO:0005925; FDR = 6.94E-23) and extracellular matrix structural constituent (GO:0005201; FDR = 1.19E-11) (Fig. 4e).

During hospitalization, serial coagulation monitoring in our patient with CILD40 and malignancy demonstrated persistent, low-grade elevations in fibrinolytic markers (D-dimer: 2.92–4.47 mg/L [normal range: 0–0.55 mg/L] and FDP: 9.16–13.43 mg/LFEU [normal range: 0–5 mg/LFEU]) (Table S2). These values remained below the critical thresholds, not warranting therapeutic anticoagulation in the absence of radiologically confirmed thrombosis or high-risk clinical features. To delineate whether the observed coagulation protein upregulation was driven by tumor burden, we performed a comparative analysis of GO terms: "blood coagulation", "hemostasis", and "platelet activation" between the proband (II-2, with malignancy) and her sister (II-1, without malignancy), and found that the abundances of key regulators were highly comparable (Fig. 4f, g), indicating that the hypercoagulable signature was a direct consequence of *DNAH9* variants, and was independent of concurrent malignancy. Proteomic analysis further corroborated ciliary involvement. A comparison of the DEPs of the proband (II-2) and her sister (II-1) with those in SCGSv2 revealed 31 shared ciliary proteins (Fig. 4h, i). This overlap was significantly enriched in critical ciliary-related biological processes, including Cilium assembly (GO:0060271; FDR = 3.11E-05), Microtubule-based process (GO:0007017; FDR = 2.05E-09) and Microtubule cytoskeleton organization (GO:0000226; FDR = 9.25E-06) (Fig. 4j, Table S3). Collectively, the plasma proteomic signatures in *DNAH9*-mutant patients provide direct biochemical evidence of the role of *DNAH9* in ciliary proteostasis, indicating a potential causal link between *DNAH9* compound heterozygosity and global ciliary dysfunction in CILD40.

### Differential Plasma Proteomic Signatures in CCDC40- and *DNAH9*-Mutant Patients with PCD

**Fig. 5:**
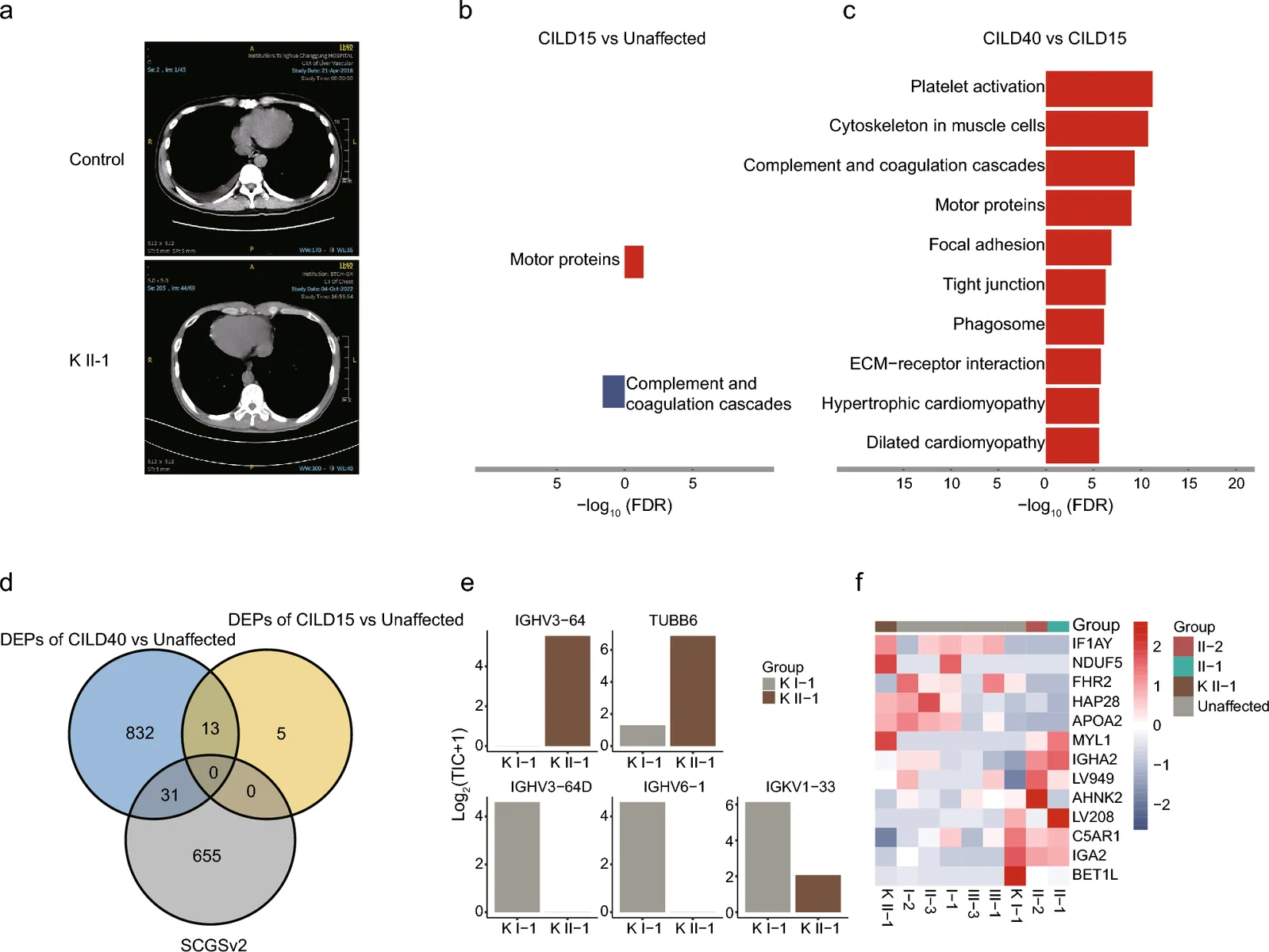
Differential plasma protein signatures in CILD15 and CILD40. **a** Enhanced chest computed tomography (CT) scan image of a patient with CILD15 and situs inversus, and of a control sample.** b** Ten most enriched KEGG pathways in CILD15 vs. unaffected. **c** Ten most enriched KEGG pathways in CILD40 vs. CILD15. **d** The Venn diagram shows DEPs in CILD40 vs. unaffected, CILD15 vs. unaffected, and the curated SCGSv2 proteome. **e** Bar plots of CILD15-specific DEPs. **f** Heatmap of the 13 common DEPs in CILD40 vs. unaffected and CILD15 vs. unaffected

Kartagener syndrome (KS) is a distinct subtype of PCD characterized by the classical triad of situs inversus, bronchiectasis, and chronic sinusitis (Casanova et al. 2006; Ciancio et al. 2015; Leigh et al. 2009). Our patient (K II-1) was diagnosed with bronchiectasis with infection (caused by *Klebsiella pneumoniae*), situs inversus (dextrocardia; Fig. 5a), and infertility. By integrating the clinical phenotypes with the pathogenic mutations identified using WES, homozygous mutations in *CCDC40* (NM_017950.4: c.1806 + 1G > T) were identified as being linked to CILD15 and as the pathogenic variants in this patient. We compared the plasma proteomic changes associated with *DNAH9* mutations in CILD40 with those in *CCDC40* mutations in CILD15 (KS). Notably, DE analysis identified only 18 DEPs in the peripheral blood proteome between the CILD15 proband and his healthy parents, including nine upregulated and nine downregulated proteins. However, 406 DEPs were identified by comparing CILD40 with CILD15, of which 246 were upregulated and 160 were downregulated (Table S4). KEGG pathway analysis revealed significant enrichment of up-regulated motor proteins (hsa04814; *q*-value = 3.4E-02) and down-regulated complement and coagulation cascades (hsa04610; *q*-value = 1.6E-02) pathways in patients with CILD15 (Fig. 5b, Table S4). In contrast to those in CILD15, the upregulated DEPs in CILD40 were closely associated with platelet activation (hsa04611; *q*-value = 2.70E-14), cytoskeleton in muscle cells (hsa04820; *q*-value = 1.56E-13), and complement and coagulation cascades (hsa04610; *q*-value = 6.21E-12) (Fig. 5c, Table S4).

To study the biological implications of these differential profiles through disrupted ciliary assembly networks, we compared the DEPs in CILD40 with the SCGSv2 gold standard, revealing 31 shared ciliary proteins between the two siblings and no shared ciliary proteins with CILD15 (Fig. 5d, Table S4). Notably, there were only five CILD15-specific non-ciliary protein DEPs, including TUBB6, IGHV3-64, IGHV3-64D, IGHV6-1, and IGHV1-33 (Fig. 5e), and their discordant expression highlighted distinct pathogenic cascades in CCDC40-deficient patients and underscored the interplay between structural ciliopathy and dysregulated immune adaptation. Surprisingly, 10 of the 13 DEPs shared between CILD15 and CILD40 exhibited divergent regulation of the two subtypes. Compared to CILD15, CILD40 showed significantly upregulated immunoglobulin components (IGA2), complement receptors (C5AR1), and stress-response factors (LV949, AHNK2), and downregulated lipid transporters (APOA2) and mitochondrial/translational regulators (NDUF5) (Fig. 5f, Table S4), which, to some extent, reflected mucosal hyperinflammation and metabolic reprogramming in CILD40.

## Discussion

PCD is a rare, genetically heterogeneous disorder characterized by impaired motile ciliary function, leading to chronic respiratory infections, organ laterality defects, infertility, and other systemic abnormalities (Leigh et al. 2019). Despite significant advances in gene discovery, the current classification of PCD relies heavily on newly identified genes, with limited integration of other phenotypic evidence for more precise subtyping (Horani et al. 2016). Although genetic testing has improved our understanding of genotype–phenotype relationships, it cannot be used in isolation, because of the high frequency of private mutations and variants of unknown significance. For example, mutations in genes such as *DNAH9* and *RSPH1* are associated with milder disease phenotypes, whereas defects in *CCNO* or *MCIDAS* are associated with more severe clinical presentations (Boon et al. 2014; Brennan et al. 2021; Pifferi et al. 2025; Wallmeier et al. 2014). Additionally, mutations in some PCD-associated genes, such as *DNAH9*, may result in normal ciliary ultrastructure or nasal NO levels, highlighting the need for complementary functional tests to identify differential phenotypes (Kuehni and Lucas 2017).

In this study, we identified novel compound heterozygous mutations in the *DNAH9* gene in two patients within a three-generation pedigree. We systematically investigated the clinical features and peripheral blood transcriptome and proteome differences between patients with CILD40 and their heterozygous family members and one unaffected relative. Notably, this study represents the first comprehensive characterization of the peripheral blood transcriptome and proteome of CILD40, revealing distinct protein profiles associated with this subtype. Additionally, we compared the proteomic signatures of CILD40 with those of CILD15 to further elucidate the diverse genotype–phenotype landscapes underlying different PCD subtypes.

*DNAH9* is an ODA type 2 dynein heavy chain protein. Motile cilia at the embryonic node are essential for establishing left-right symmetry. Dysfunction of cilia in the embryonic node disrupts normal left-right organ patterning, leading to randomized organ distribution and resulting in situs inversus in 40%–50% of affected individuals and heterotaxy in a smaller subset of patients (Kennedy et al. 2007; Lucas et al. 2014; Nakhleh et al. 2012; Raidt et al. 2015; Werner et al. 2015). Currently, data summarizing the situs abnormalities caused exclusively by *DNAH9* mutations are limited. Through a comprehensive search of the OMIM database (Amberger et al. 2015) and a review of the existing literature, we identified 31 cases involving either monoallelic or biallelic *DNAH9* mutations, with 72.41% (21/29) of these cases presenting with situs abnormalities. The predominant phenotypic features associated with *DNAH9* mutations are laterality defects. In our analysis of the three-generation pedigree, two affected individuals exhibited distinct situs abnormalities, one with complete situs inversus and the other with heterotaxy, including the absence of a retrohepatic inferior vena cava, polysplenia syndrome, bile duct dilation, and congenital pancreatic hypoplasia. These findings highlight the diverse phenotypic outcomes of *DNAH9* mutations, ranging from complete organ reversal to a complex heterotopic presentation.

We further explored the peripheral blood transcriptional landscape of CILD40 by focusing on the intersection of DEGs shared between the two siblings and cross-referencing them with the SysCilia Gold Standard v2 ciliary gene set, thereby isolating cilia-specific alterations from tumor-related expression changes. Despite these variations, both sisters exhibited relatively consistent ciliary gene signatures associated with CILD40. Ciliary dysfunction disrupts cytoskeletal organization, cell-cell adhesion, and intracellular signaling, leading to widespread abnormalities in cell motility and immune responses (Cockx et al. 2021, 2017a, 2017b, 2018; Lu et al. 2023). Dysregulated immune responses have also been observed in the blood transcriptomes of patients with CILD40. Consistent with the limited number of single-cell studies reporting transcriptomic alterations in nasal mucosal ciliated cells of patients with PCD (Brody et al. 2025; Koenitzer et al. 2024), our findings support systemic manifestations of structural ciliary defects, suggesting that blood transcriptomes may reflect tissue-level ciliary transcriptional signatures.

Plasma cellular proteins are key determinants of phenotypes and their precise regulation is crucial for maintaining homeostasis and driving pathogenesis. Our proteomic analysis of peripheral blood further revealed highly similar protein expression profiles between patients with CILD40 and identified dysregulated immune and coagulation pathways. While direct studies on hemostatic alterations in patients with PCD remain scarce, mechanistic links have emerged through dysregulated inflammation, complement activation, and platelet aggregation. Recent single-cell studies of PCD have demonstrated that ciliary ultrastructural defects elicit cell-intrinsic inflammatory and stress programs in airway epithelia independent of environmental triggers, thereby providing a plausible upstream driver for systemic signals detected in the blood (Koenitzer et al. 2024). Consistent with this framework, loss of DNAH9 function specifically perturbs the distal ODA assembly and impairs distal axonemal bending, leading to defective mucociliary clearance and epithelial stress, which are upstream lesions repeatedly observed in human cohorts with DNAH9 mutations and models (Loges et al. 2018). To anchor our blood-based findings to ciliopathy biology, rather than nonspecific inflammation, we cross-referenced DEGs/DEPs to the SYSCILIA Gold Standard v2 compendium and observed the enrichment of first-order ciliary components and processes, consistent with perturbed ciliary proteostasis in CILD40. At the systemic level, several leading DEPs fall on the canonical platelet adhesion/activation and complement–coagulation crosstalk axes, which mechanistically link epithelial injury to a thrombo-inflammatory plasma phenotype. Specifically, the VWF–GPIb–αIIbβ3 (ITGA2B/ITGB3) module, together with fibronectin (FN1) and SRC-family kinases, constitutes a well-defined outside-in signaling pathway that stabilizes platelet adhesion under shear and amplifies aggregation. Perturbation along this axis explains the enrichment of platelet/coagulation terms (Durrant et al. 2017). Moreover, the bulk RNA sequencing results of primary nasal cells from three patients with PCD who did not undergo single-cell RNA sequencing tests demonstrated enrichment of the term 'Support of platelet aggregation by Eph kinase and ephrins' (Koenitzer et al. 2024). Collectively, these limited, but convergent findings provide compelling biological plausibility for *DNAH9* variant–induced plasma proteomic signatures of dysregulated coagulation, although they warrant validation in dedicated *in vivo* models.

Although more than 50 PCD genes have been identified, studies of PCD-related gene variants have primarily focused on uncovering novel genotype–phenotype correlations in patient cohorts. Moreover, systematic comparative studies of the mechanisms underlying various PCD subtypes are lacking, despite their importance in understanding the disease spectrum. We initially performed a comparative proteomic analysis between patients with CILD15 and the CILD40 family group. Notably, compared with healthy controls, patients with CILD15 showed far fewer DEPs than patients with CILD40. To some extent, the plasma proteomic disparities between CILD40 and CILD15 epitomize how initial molecular lesions dictate distinct pathophysiological trajectories. The profound axonemal collapse in CILD40 drives sustained epithelial injury, unleashing systemic inflammation and metabolic derangement. Conversely, the confined dynein defect in CILD15 permits localized adaptive responses that manifest as TUBB6-mediated cytoskeletal compensation and antigen-specific humoral immune responses. This mechanistic stratification also provides a preliminary indication that PCD subtypes are not merely clinical variants but discrete entities with differential plasma signatures and therapeutic implications. Consequently, while the current literature and population-scale datasets, such as the UK Biobank (with only one PCD patient in its OLINK plasma proteomic cohort under ICD-10 code Q34.8), remain underpowered for subtype analyses, future large-scale PCD cohort studies should prioritize plasma proteomic divergence profiling to delineate subtype-specific pathomechanisms and therapeutic vulnerabilities.

A significant limitation in studying the mechanisms of rare diseases is the lack of animal models that accurately recapitulate patient genotypes. Although *DNAH9*-knockdown (KD) mice display phenotypes consistent with PCD, including disrupted ciliary morphology, reduced ciliary beat frequency, and impaired lung function (Zheng et al. 2022), these models do not fully recapitulate the complex clinical features of CILD40. Specifically, patients with CILD40 exhibit situs abnormalities, subtle upper respiratory symptoms, infections, and male infertility, whereas mouse models may not fully represent these phenotypic nuances. For example, the ciliary defects observed in *DNAH9*-KD mice are primarily localized to the distal portion of the cilia, a fingding that is consistent with the subtle defect seen in patients with CILD40. However, the mouse model does not fully capture the spectrum of clinical manifestations. Therefore, the establishment of more refined animal models, particularly those reflecting the specific genotypes of patients with CILD40, is essential to validate our findings and explore organ-specific functional alterations.

Our study has several limitations inherent to the investigation of rare diseases, such as PCD. First, our PCD diagnosis was based primarily on the molecular genetic findings and clinical features defined for CILD40 in OMIM, as both sisters declined nasal NO measurements, HSVM, and TEM testing. The lack of patient-derived functional assays may limit the diagnostic accuracy and increase the risk of false positives. In future work, on suspected PCD patients with mild upper respiratory symptoms and infections, TEM, HSVM, and nasal NO measurements will be prioritized to facilitate a precise diagnosis using patient-derived samples (e.g., nasal epithelial and bronchial epithelial samples). Second, the limited availability of clinical samples, particularly tissue specimens, precludes further experimental validation and necessitates reliance on peripheral blood samples for phenotypic analysis. Third, the rarity of CILD40 significantly restricted the sample size, thereby limiting the generalizability of our findings. We recognize that our case-to-control ratio remains limited, with only two affected individuals versus five controls. This imbalance may inflate type I errors and limit generalizability. However, the rarity of CILD40 and the ethical constraints on invasive sampling restricted cohort expansion. Our use of stringent FDR correction and the inclusion of multiple unaffected family members mitigated these limitations. In addition, the plasma proteomics results were derived from a discovery LC–MS/MS workflow and lacked orthogonal cross-validation, which constrains the interpretation of analytical specificity, quantitative accuracy, and external validity. Immunoassays (e.g., enzyme-linked immunoassays or western blotting) may help confirm target presence and the direction of change in an analytically independent modality, strengthening biological credibility, while targeted mass spectrometry (SRM/PRM with stable-isotope standards) may verify the specificity, quantitative linearity, and limits of detection/quantification for selected candidates. Replication in independent cohorts would help evaluate effect-size stability and external validity. In the absence of such elements, pathway-level interpretation should be provisional.

Future studies will seek to validate these findings in larger, independent PCD cohorts using functional assays. Despite these constraints, blood-based phenotypic studies remain crucial for elucidating systemic alterations in rare diseases, as they offer a non-invasive approach to capture generalized molecular changes. To achieve a more precise classification and management of PCD, future studies should focus on integrating genetic findings with comprehensive phenotypic data, including tissue-specific analyses and functional assays. This approach will help elucidate the full spectrum of PCD phenotypes and facilitate the development of personalized management plans based on genotype–phenotype correlations.

In summary, we identified novel compound heterozygous mutations in *DNAH9*, NM_001372.4:c.308del (p.F103Sfs*31), and NM_001372.4:c.11845G >A (p.A3949T), in a three-generation pedigree. By integrating transcriptomic and proteomic analyses, we delineated the molecular mechanisms underlying disease pathogenesis and their association with observed phenotypic manifestations. Additionally, our comparative proteomic analysis of CILD15 and CILD40 provided further insights into the molecular distinctions between these PCD subtypes. Collectively, our findings enhance our understanding of PCD, particularly CILD40, and highlight the molecular heterogeneity that contributes to its diverse clinical presentations.

## Supplementary Information

Below is the link to the electronic supplementary material.

Supplementary file1 (PDF 3588 KB)

Supplementary file2 (XLSX 637 KB)

Supplementary file3 (XLSX 244 KB)

Supplementary file4 (XLSX 10 KB)

Supplementary file5 (XLSX 145 KB)

## Data Availability

Bulk RNA sequencing and plasma proteomics data were deposited in the Genome Sequence Archive for Humans at the China National Center under the accession numbers HRA012058 (RNA sequencing) and OMIX010797 (proteomics).

## Abbreviations

PCD: Primary ciliary dyskinesia
OMIM: Online Mendelian Inheritance in Man
***DNAH9***: *Dynein axonemal heavy chain 9*
CILD40: Primary ciliary dyskinesia 40
IDA: Inner dynein arm
ODA: Outer dynein arm
DNAH5: Dynein axonemal heavy chain 5
DNAH11: Dynein axonemal heavy chain 11
TEM: Transmission electron microscopy
SNV: Single nucleotide variant
Indels: Insertion-deletion variants
CNV: Copy number variation
NO: Nitric oxide
CTA: Computed tomography angiography
MRCP: Magnetic resonance cholangiopancreatography
WT: Wild-type
PCA: Principal component analysis
DEGs: Differentially expressed genes
SCGSv2: SYSCILIA Gold Standard v2
DEPs: Differentially expressed proteins
PPI: Protein–protein interaction
KD: Knockdown
WES: Whole-exome sequencing
DE: Differential expression
FC: Fold change
GO: Gene Ontology
KEGG: Kyoto Encyclopedia of Genes and Genomes
TIC: Total ion current
