# Supplementary material for: Comprehensive Transcriptomic and Proteomic Profiling of CILD40 with Novel Compound Heterozygous Mutations of DNAH9
Source: Phenomics. 2026 Jul 28;6(5):611–34. doi: 10.1007/s43657-026-00318-0 (PMC13743698; doi:10.1007/s43657-026-00318-0)
Supplement: Supplementary file 1 [file 43657_2026_318_MOESM1_ESM.pdf]

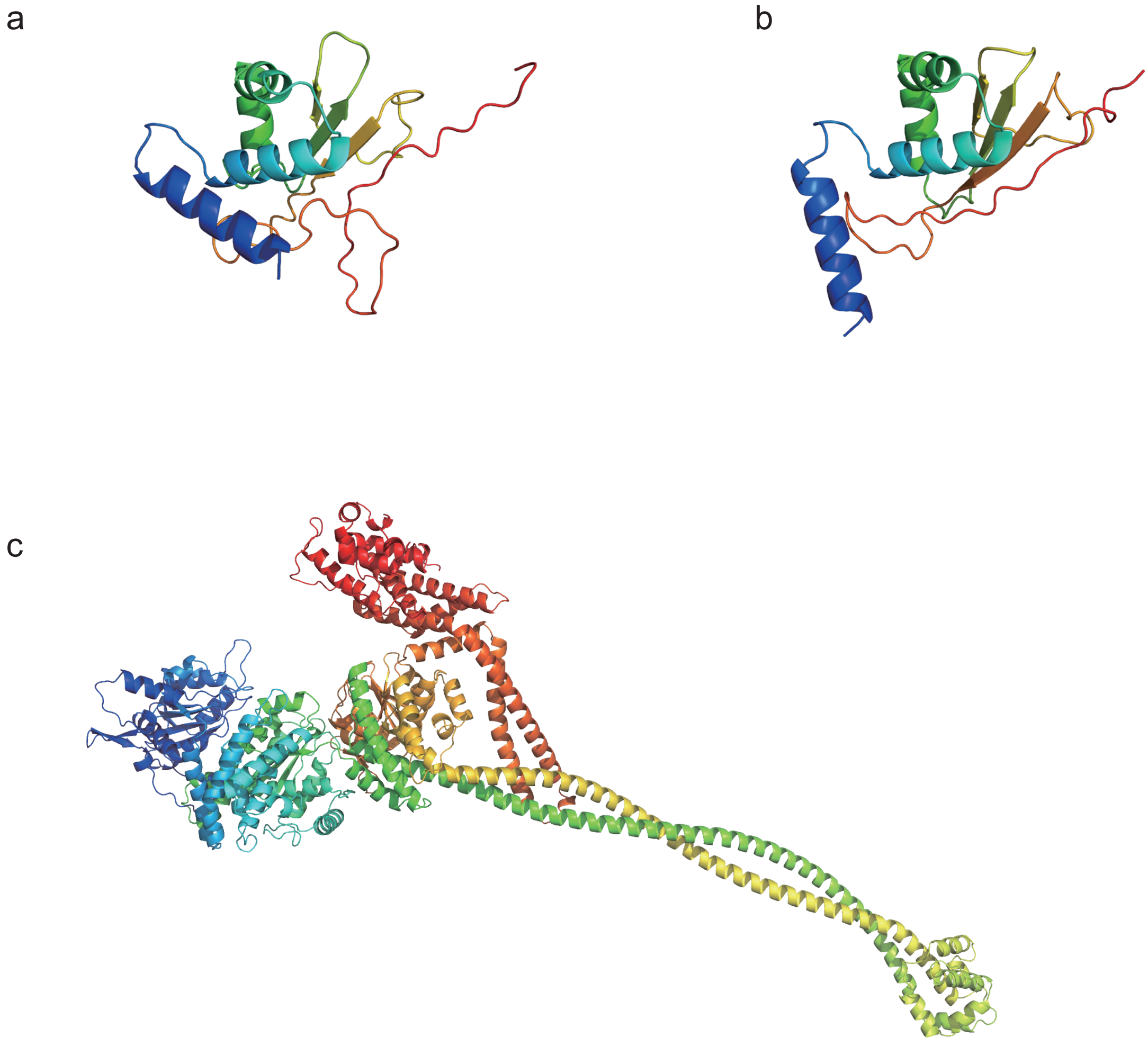
a
b
c
